# Dysglycemia associations with adipose tissue among HIV-infected patients after 2 years of antiretroviral therapy in Mwanza: a follow-up cross-sectional study

**DOI:** 10.1186/s12879-017-2209-z

**Published:** 2017-01-30

**Authors:** George PrayGod, John Changalucha, Saidi Kapiga, Robert Peck, Jim Todd, Suzanne Filteau

**Affiliations:** 1 Mwanza Research Centre, National Institute for Medical Research, Box 1462, Mwanza, Tanzania; 2 Mwanza Intervention Trials Unit, Mwanza, Tanzania; 3 Weill Bugando School of Medicine, Mwanza, Tanzania; 4 Weill Cornell Medical College, New York, USA; 5 London School of Hygiene and Tropical Medicine, London, UK

**Keywords:** Pre-diabetes, Diabetes, HIV, Antiretroviral therapy, Body composition, Anthropometry, Africa

## Abstract

**Background:**

Data on the burden of dysglycemia among HIV-infected patients on antiretroviral therapy (ART) in Africa are limited. We determined the prevalence of pre-diabetes and diabetes among HIV-infected patients who started ART when malnourished 2 to 3 years previously and investigated the association of dysglycemia with body composition.

**Methods:**

Malnourished (body mass index (BMI) < 18.5 kg/m^2^) HIV-infected patients who were enrolled in the Nutritional Support for Africans Starting Antiretroviral Therapy (NUSTART) trial from 2011 to 2013 were followed-up from March to August 2015. Anthropometric, fat mass and fat-free mass by bioelectrical impedance, and C-reactive protein (CRP) data were collected at baseline and follow-up. At follow-up, we defined fasting glucose of 6.1–6.9 mmol/L as impaired fasting glucose (IFG) and 2-h oral glucose tolerance test (OGTT) glucose of ≥7.8 to <11.1 mmol/L as impaired glucose tolerance (IGT). Both of these were considered pre-diabetes. Fasting glucose of ≥7.0 mmol/L or impaired glucose tolerance of ≥11.1 mmol/L was defined as diabetes mellitus. The relation of pre-diabetes and diabetes with body composition was assessed using logistic regression.

**Results:**

Two hundred seventy-three (57%) of 478 patients who were alive at trial conclusion were followed-up. The mean age was 41.5 (SD 9.8) years and 65.2% (178) were females. The mean follow-up BMI was 19.9 (SD 2.8) kg/m^2^, 12 (4.4%) were either overweight or obese, and 61 (22.3%) patients had pre-diabetes or diabetes. In multiple regression, upper tertiles of baseline hip circumference (OR: 0.41, 95% CI: 0.2, 0.8) and fat mass index (OR: 0.20 (0.1, 0.5), and upper tertiles of follow-up waist circumference (OR: 0.22 (0.1, 0.5), BMI (OR: 0.32 (0.1, 0.7), fat mass index (OR: 0.19 (0.1, 0.5) and the middle tertile of follow-up fat-free mass (OR: 0.36, 95% CI: 0.1, 0.8) were associated with lower risk of pre-diabetes and diabetes (*P* < 0.05 for all). Baseline and follow-up CRP were not predictors.

**Conclusions:**

Low rather than high measures of adipose tissue were associated with increased risk of pre-diabetes and diabetes. Additional studies are needed to further investigate the role of body composition and control of glucose metabolism in the pathogenesis of diabetes among persons living with HIV in Africa.

**Electronic supplementary material:**

The online version of this article (doi:10.1186/s12879-017-2209-z) contains supplementary material, which is available to authorized users.

## Background

Diabetes mellitus is an emerging major public health problem in Africa [1] which has been associated with epidemiologic nutritional transition, globalization, and emergence of infections like HIV [1, 2]. Data from developed countries suggest that HIV-infected patients have a higher risk than HIV-uninfected people of developing diabetes and other non-communicable diseases [3, 4]. The excess diabetes risk is probably related to multiple factors including HIV-associated inflammation, the use of some antiretroviral therapy (ART) regimens, and body composition changes associated with HIV and ART [5, 6]. As a result, HIV-infected populations may develop diabetes at a younger age and may have a higher mortality if management is not optimal, as may be the case in resource-limited countries of Sub-Saharan Africa (SSA).

HIV immune activation and inflammation are hallmarks of HIV pathogenesis and increase the risk of non-communicable diseases including diabetes [7]. In guinea pigs, pro-inflammatory cytokines such as tumor necrosis factor (TNF)-alpha and interleukin 6 (IL-6) produced during inflammation promote the release of free fatty acids from adipose tissue which may reduce insulin action on skeletal muscles resulting in dysglycaemia and diabetes mellitus [8]. In SSA where HIV patients have multiple opportunistic infections, including tuberculosis (TB), and often start ART when they are very sick and have severe or chronic inflammation, the risk is likely to be higher, but data are limited. The use of some ART regimens may increase diabetes risk [3, 4] through mitochondrial toxicity of skeletal muscle and adipose tissues. The problem may be greater in SSA than in high income countries since older ART drugs which have higher levels of toxicity are often still used in SSA.

Furthermore, HIV-infected patients who may have started ART with low body mass index (BMI) [9] will rapidly regain weight once on ART; however, this weight increment may mainly comprise adipose tissue rather than lean mass since persistent inflammation limits regain of lean mass [10, 11] This excessive increment in adipose tissue relative to lean mass may increase the risk of diabetes mellitus.

Most of the data to-date on HIV and diabetes are from high-income countries, and data in SSA are few and inconsistent [12–14]. Because of differences in genetic composition as well as environmental factors including high burden of infectious diseases in resource-limited settings, data from high-income countries cannot be extrapolated and reliably used to improve quality of diabetes care among HIV patients in SSA. It is therefore crucial that HIV programmes in SSA, where the majority of HIV-infected patients in the world reside, get data on the magnitude of and risk factors for diabetes among HIV-infected patients to help plan for prevention and treatment interventions. Therefore, we followed-up HIV-infected patients who were malnourished when they enrolled 2–3 years previously in the Nutritional Support for Africans Starting Antiretroviral Therapy (NUSTART) trial [15]. We determined in these patients the prevalence of pre-diabetes and diabetes and investigated associations with anthropometric and body composition measurements on the background of very high HIV-associated inflammation [16].

## Methods

### Study design and setting

This was a follow-up cross-sectional study conducted from March to August 2015 at the National Institute for Medical Research (NIMR), Mwanza, in north-western Tanzania. Participants were eligible if they had been recruited in the NUSTART trial and provided informed consent for this follow-up study which included consent to use their NUSTART data for this study. NUSTART was a phase III randomized controlled trial testing the efficacy of vitamins and minerals in a lipid-based supplement on morbidity and mortality of malnourished (BMI < 18.5 kg/m^2^) HIV-infected patients starting ART and was conducted in Lusaka, Zambia and Mwanza, Tanzania from August 2011 to December 2013 [15]. Enrolment and exclusion criteria and other study details have been published previously [15]. The current study sought follow-up of all participants who completed the NUSTART study in Mwanza (12 weeks post-ART initiation), 2–3 years later.

### Study participants follow-up strategy

To maximise follow-up of participants, all patients who were known to be alive at the end of the NUSTART trial were telephoned several times to introduce them to the follow-up study, to determine if they were interested, and to make an appointment. Potential participants who were not reachable by phone were visited at home and invited to join the study. If patients were not available to respond to the call or talk with the study tracer during home visits, we requested people responding to the call or those found in participants’ homes to provide information on participants’ vital status and if they had travelled or relocated permanently from the Mwanza city. Patients given clinic visit appointments were requested to come to the NIMR Research Clinic in Mwanza City at 8:00 am after an overnight fast of at least 8 h to receive further study information, and for consenting and study procedures.

### Data collection

Before study data collection began, research assistants were recruited and trained on standard operating procedures for consenting, questionnaire administration, blood sampling, and glucose testing and interpretation.

### Questionnaire data

Questionnaire data were collected using structured interviews. Based on WHO STEPS manual questionnaire [17], information on demography, education level, occupation, religion, marital status, smoking, alcohol use, fruit and vegetable consumption, and level of physical activity. We used show cards in the WHO STEPS manual [17] to assist data collection. For alcohol and smoking, the key questions were whether patients had ever taken alcohol and/or smoked and if they were current alcohol drinkers and/or smokers. Taking alcohol in the past 30 days was considered current alcohol drinking and patients who reported to have ever smoked and had not stopped smoking at the time of the study were considered current smokers. Fruit and vegetable intake was assessed based on consumption history in the past 12 months. To help patients remember what they usually eat and amounts, we prepared a list of commonly available fruits and vegetables in Mwanza and provided a container for them to estimate the quantity of fruits and vegetables they are taking on days they reported to be eating fruits and vegetables. Using this information, a trained research assistant converted the quantities ingested into servings; with one serving weighing approximately 80 g. We also collected information on ART use history and adherence, TB treatment history from NUSTART enrolment, and symptoms of diabetes. Information on ART use history was verified through patients’ ART cards and ART clinic records.

### Glucose testing

Before glucose testing, the research assistant asked patients if they had taken any food except water (fasted) for at least 8 h before visiting the clinic. Those fasting were finger pricked to provide blood for glucose measurement using a Hemocue machine (Hemocue AB, Angelholm, Sweden). After fasting glucose assessment, all patients were subjected to a 2-h oral glucose tolerance test (OGTT) where they were provided with 82.5 g of dextrose monohydrate (equivalent to 75 g of glucose anhydrous) diluted in 250 mls of drinking water to drink within 5 min. The OGTT glucose assessment was done 2 h later. Based on WHO guidelines [18], patients whose fasting glucose ranged between 6.1 and 6.9 mmol/L were diagnosed with impaired fasting glucose (IFG) whereas those with OGTT glucose level of ≥7.8 to <11.1 were diagnosed with impaired glucose tolerance (IGT). Patients with either IFG or IGT were classified as having pre-diabetes, a condition associated with higher risk of developing diabetes mellitus [19]. Diabetes mellitus was defined as fasting glucose of ≥7.0 mmol/L or impaired glucose tolerance of ≥11.1 mmol/L.

### Blood for C-reactive protein (CRP)

Venous blood samples were collected and centrifuged for serum CRP testing by ELISA (AssayPro, St. Charles, MO, USA) in the NIMR, Mwanza immunology laboratory.

### Retrieval of NUSTART data

As part of this study, we retrieved and used NUSTART trial data. Data used include baseline socio-economic status, trial enrolment date, ART start date, baseline CD4 count, TB treatment history at trial enrolment, baseline log-transformed CRP data, baseline fat and fat free mass indices measured by bioelectrical impedance analysis (BIA), and baseline waist and hip circumferences.

### Follow-up anthropometry and body composition data

During the current study, we determined anthropometric measurements using standardized methods. While barefoot and with minimal clothing, weight of the patient was determined to the nearest 0.1 kg using a digital scale (Seca, Germany) and height was measured to the nearest 0.1 cm using a stadiometer fixed to the office wall (Seca, Germany). Waist and hip circumferences were measured using non-stretchable tape (to 1 mm). Anthropometric measurements were taken in triplicate and medians were used during analysis. Also similar to NUSTART baseline [10], participants underwent BIA to estimate fat mass and fat-free mass (Tanita BC418, Tokyo, Japan). Since internal equations in the BIA Tanita machine were derived from studies of predominantly non-malnourished populations, and may not account for differences in hydration status and other factors present in malnourished, HIV-infected individuals [20], plethysmography data (BodPod 2007A, Life Measurement Instruments/COSMED, California, USA) collected at NUSTART baseline in a sub-sample of Zambian patients were used to adjust the BIA calculations for baseline and follow-up data to account for changes in body composition unique to this patient population. The adjustment was done using BodPod since BodPod data are more likely to be accurate and precise than BIA data in malnourished populations [21]. In the calibration, regression analysis was used to develop an optimal equation to predict BodPod fat-free mass with BIA Impedance index (height squared/whole body impedance), weight, height and sex as dependant variables. Based on this, the final adjusted BIA fat-free mass data were compiled using this equation: Fat-free mass = −5.951 + (height squared/whole body impedance × 0.292) - (female sex × 1.345) + (weight × 0.324) + (height × 0.128) (Wells JCK and Woodd S, unpublished data). The method used was similar to that used in validating BIA against Bodpod among Ethiopian infants [22]. Subsequently fat mass was computed as: weight minus fat-free mass. From BIA fat and fat-free mass, and height measurements, fat mass index was computed as fat mass (kg)/[height (m^2^)], and fat-free mass index as fat-free mass (kg)/[height (m^2^)] [23]. We used fat mass and fat-free mass indices instead of fat and fat-free mass to remove the effect of variability in size.

### Sample size

The sample size for this study was based on the conventional formula for the confidence interval around an Odds Ratio for the increased odds of pre-diabetes and diabetes (main outcome) [24]. Based on previous studies, we anticipated that at least 20% of ART patients would have a high level of inflammation, and 20% would have excessive deposition of adipose tissue (the two main risk factors), and that 10% of the patients would have pre-diabetes or diabetes. We aimed to trace 60% of 478 of surviving patients from the NUSTART study, and with a sample size of 50 patients with pre-diabetes or diabetes and 200 patients without, the study would have 90% power to detect an Odds Ratio of 4 as significant at the 5% level.

### Data management and statistics

Data were double entered into OpenClinica database and analysed in STATA version 12 (Station College, Texas, USA). Cohort characteristics were presented as means or percentages as appropriate. Comparisons of followed-up patients versus those not followed-up (either died or lost to follow-up) were done using *t*-test and oneway ANOVA for continuous variables and chi-squared test for categorical variables. For continuous variables with >2 comparison groups, Bonferroni post hoc test was used to determine difference between group pairs after oneway ANOVA was done.

Combined pre-diabetes and diabetes was the outcome variable whereas baseline and follow-up body composition parameters (fat and fat-free mass indices), BMI, waist and hip circumferences, and log-transformed baseline and follow-up CRP were considered the main predictor variables. Demography, socio-economic status, TB treatment at NUSTART trial enrolment, TB treatment history at follow-up, smoking, alcohol drinking, fruit and vegetable intake, and ART regimens used were included in models as potential confounders. Univariate logistic regression was conducted for all predictor variables and potential confounders whose effect sizes were significant at *P* < 0.10 were adjusted for in multiple logistic regression models assessing the relationship between the outcome variable and main predictor variables. To avoid the effect of multicollinearity, we included only one anthropometric or body composition explanatory variable in these models at a time. To enhance clarity in data presentation, main analysis included anthropometric and body composition measurements as tertiles rather than continuous variables. In supplementary analyses we included anthropometric and body composition measurements in a continuous scale as predictor variables and assessed the role of body composition changes on the study outcome. The effect sizes were presented as odds ratios (OR) with 95% confidence intervals. *P* values <0.05 indicated significant differences.

## Results

### Characteristics of followed-up patients compared to those not followed-up

**Fig. 1:**
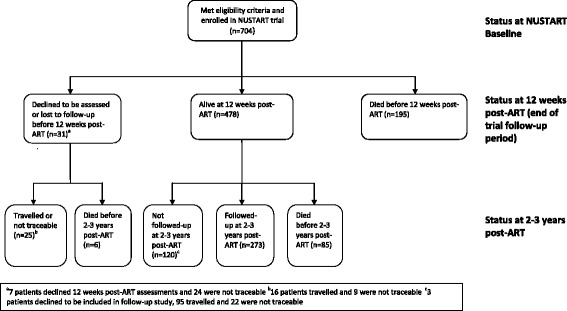
Participant flow chart

Figure 1 shows participants flow from NUSTART trial enrolment until the current study. Of 704 patients enrolled in NUSTART trial, 478 patients were alive at 12 weeks ART, i.e., the end of NUSTART trial follow-up, 273 (38.8%) were followed-up 2 to 3 years later, 286 (40.6%) had died either during or after NUSTART, and 145 (20.6%) were either lost to follow-up, had travelled out of Mwanza city or declined to participate. Baseline measures of both BMI and fat mass index were lower among patients who died before follow-up compared to those followed-up and there were differences among the three groups in sex, education attainment, CD4 count, and TB treatment history at NUSTART enrolment (Table 1).

**Table 1:** Comparison of NUSTART recruitment characteristics of patients followed-up at 2–3 years post-ART initiation versus those not followed-up (due to loss to follow-up, lack of data or death)^a^

	Followed-up at 2–3 years post ART (*n* = 273)	Lost to follow-up or died before 2–3 years-post ART (*n* = 431)		*P*-value^b^
		Lost to follow-up (*n* = 145)	Died (*n* = 286)	
Age at baseline, year	38.9 (9.7)	35.9 (9.7)	38.3 (9.9)	0.01^e^
Female, n (%)	178 (65.2)	76 (52.4)	133 (45.6)	<0.0001
Marital status, n (%)^c^				
Married	122 (44.7)	48 (33.3)	114 (39.9)	0.16
Widow/widower	38 (13.9)	17 (11.8)	40 (14.0)	
Divorced/separated	94 (34.4)	59 (41.0)	96 (33.5)	
Single	19 (7.0)	19 (13.2)	34 (11.9)	
Lives with partner	0 (0.0)	1 (0.7)	2 (0.7)	
Occupation, n (%)^c^				
Salaried	6 (2.2)	8 (5.6)	10 (3.5)	0.16
Self-employed	237 (86.8)	119 (82.6)	251 (87.8)	
Housewife	19 (7.0)	6 (4.1)	10 (3.5)	
Student	2 (0.7)	3 (2.1)	1 (0.3)	
Unemployed	9 (3.3)	8 (5.6)	14 (4.9)	
Education level, n (%)^c^				
None	81 (29.6)	31 (21.5)	82 (28.7)	0.01
Primary	176 (64.5)	104 (72.2)	192 (67.1)	
Secondary	16 (5.9)	5 (3.5)	11 (3.9)	
University/Tertiary	0 (0)	4 (2.8)	1 (0.3)	
Social economic status, n (%)				
Lowest	58 (21.3)	19 (13.1)	64 (22.4)	0.10
Low	64 (23.4)	24 (16.6)	62 (21.7)	
Middle	54 (19.8)	28 (19.3)	50 (17.5)	
High	47 (17.2)	39 (26.9)	55 (19.2)	
Highest	50 (18.3)	35 (24.1)	55 (19.2)	
Fat mass index (kg/m^2^)^d^	2.9 (0.8)	2.7 (0.8)	2.5 (0.8)	<0.0001^f^
Fat-free mass index (kg/m^2^)^d^	14.0 (0.9)	14.1 (0.8)	13.9 (1.0)	0.14
Body mass index (kg/m^2^)	16.7 (1.2)	16.7 (1.2)	16.2 (1.5)	0.0001^g^
CD4 count (cells/μL)	127 (99.7)	116 (102.8)	113 (106.8)	0.29
Tuberculosis treatment, n (%)	63 (23.1)	49 (33.8)	48 (16.8)	<0.0001

^a^Data are mean ± SD unless specified as n (%)

^b^comparison done by oneway ANOVA test for continuous outcomes and chi-squared test for categorical outcomes

^c^1 patient missing in the lost-to follow-up group

^d^25 patients in the followed-up group, 16 in the lost to follow-up group, and 68 in the died group missing

^e^Bonferroni test showed differences between the followed-up group and the lost to follow-up group (*P* = 0.009) and the lost to follow-up group and the died group (*P* = 0.049)

^f^Bonferroni test showed differences between the followed-up group and the died group (*P* < 0.0001)

^g^Bonferroni test showed differences between the followed-up group and the died group (*P* < 0.0001) and the lost to follow-up group and the died group (*P* < 0.0001)

### Characteristics of followed-up patients

The 273 patients followed up were seen 31 months (range 21 to 42) from NUSTART recruitment. As seen in Table 2, among these patients, the mean follow-up BMI was 19.9 (SD 2.8) kg/m^2^, 12 (4.4%) were either overweight or obese, 11 (4%) were current smokers, 31 (11.4%) were current alcohol drinkers, 113 (41.4%) had adequate (≥5 servings per day) fruit and vegetable consumption, 260 (95.2%) reported they were currently on ART and 209 (76.6%) patients spent >75 min in vigorous physical activity per week. On average these patients had been on ART for a total of 30 months (range 20 to 41) and on the current ART regimens for 19 months (range 16.5 to 41). Regarding dysglycaemia, 61 (22.3%) patients had either pre-diabetes or diabetes. Those with pre-diabetes and diabetes had lower body weight, fat mass index, BMI, and waist and hip circumferences compared to those with no pre-diabetes and diabetes, but there were no differences in clinical presentation (Additional file 1: Table S1). Among the followed-up patients, the mean fat mass index and fat-free mass index increased from 2.8 kg/m^2^ and 14.0 kg/m^2^ at baseline by 2.0 kg/m^2^ and 1.0 kg/m^2^ by 2–3 years follow-up (*P* <0.0001 for all).

**Table 2:** Characteristics at follow-up of 273 patients included in the study^a^

Age at follow-up, year	41.5 (9.8) (Range 21–75)
Religion	
Christian	225 (82.4)
Muslim	34 (12.5)
No religion	14 (5.1)
Smoking status, n (%)	
Never	197 (72.2)
Past	65 (23.8)
Current	11 (4.0)
Alcohol drinking, n (%)^b^	
Never	108 (39.7)
Past	133 (48.9)
Current	31 (11.4)
Fruits and vegetables consumption per day, n (%)	
1–2 servings	81 (29.7)
3–4 servings	79 (28.9)
≥ 5 servings	113 (41.4)
Time spent in vigorous physical activity per week (minutes), n (%)	
Inadequate (<75 min)	64 (23.4)
Adequate (≥75 min)	209 (76.6)
Weight (kg)	52.8 (8.5)
Body mass index (kg/m^2^)	19.9 (2.8)
Nutritional status according to BMI categories, n (%)	
Undernourished (BMI < 18.50 kg/m^2^)	73 (26.7)
Normal weight (BMI 18.50–24.99 kg/m^2^)	188 (68.9)
Overweight (BMI 25.00–29.99 kg/m^2^)	10 (3.7)
Obese (BMI ≥ 30.00 kg/m^2^)	2 (0.7)
Fat mass index (kg/m^2^)^c^	4.3 (2.4)
Fat-free mass index (kg/m^2^)^c^	15.5 (1.6)
Current antiretroviral therapy regimen, n (%)^d^	
AZT + 3TC + EFV	41 (15.8)
AZT + 3TC + NVP	84 (32.3)
TDF + FTC + EFV	70 (26.9)
TDF + 3TC + EFV	65 (25.0)
Pre-diabetes, n (%)^e^	57 (20.8)
Diabetes Mellitus, n (%)	4 (1.5)
Pre-diabetes (IFG + IGT) and Diabetes Mellitus, n (%)	61 (22.3)

*AZT* Zidovudine, *3TC* Lamivudine, *EFV* Efavirenz, *NVP* Nevirapine, *TDF* Tenofovir, *FTC* Emtricitabine

^a^Data in mean(sd) or n (%)

^b^272 patients included in analysis

^c^269 patients included in analysis

^d^260 patients included in analysis

^e^Impaired Fasting Glucose (IFG) or Impaired Glucose Tolerance (IGT)

### Predictors of pre-diabetes and diabetes

In univariate logistic regression (Table 3), higher socioeconomic status, higher fruit and vegetable intake, and history of TB treatment since NUSTART recruitment were associated with lower probabilities of having pre-diabetes and diabetes; similar trends towards lower diabetes and pre-diabetes were seen in patients who reported to be on TB treatment at NUSTART baseline and those who were current alcohol drinkers. Baseline and follow-up CRP, baseline CD4 count, and current ART regimens were not predictors. Higher baseline hip circumference and fat mass index, and higher follow-up waist and hip circumferences, and body mass and fat mass indices were associated with lower probabilities of having pre-diabetes and diabetes (*P* < 0.005 for all).

**Table 3:** Univariate analysis of socio-demography, alcohol drinking, smoking, fruit and vegetable intake, physical activity, and body composition as predictors for pre-diabetes and diabetes at 2 to 3 years post-ART

	PD/DM+^a^ (n = 61)	PD/DM-^b^ (n = 212)	Odds Ratio (95% CI)	*P*-value
	n(%) or mean (sd)	n(%) or mean (sd)		
Age at follow-up, years	41.8 (7.9)	41.4 (10.3)	1.00 (0.9–1.0)	0.76
Sex				
Male	24 (39.3)	71 (33.5)	Reference	0.40
Female	37 (60.7)	141 (66.5)	0.78 (0.4, 1.4)	
Marital status				
Married/cohabiting	26 (42.6)	104 (49.1)	Reference	0.59
Widow/widower	9 (14.7)	36 (16.9)	1.00 (0.4, 2.3)	
Separated/divorced	22 (36.1)	57 (26.9)	1.54 (0.8, 2.9)	
Single	4 (6.6)	15 (7.1)	1.07 (0.3, 3.5)	
Occupation				
Self-employed	51 (83.6)	181 (85.4)	Reference	0.41
Salaried	3 (5.0)	12 (5.7)	0.89 (0.2, 3.2)	
Housewife	1 (1.6)	9 (4.2)	0.39 (0.05,3.2)	
No employment/other	6 (9.8)	10 (4.7)	2.13 (0.7, 6.1)	
Highest education level				
Never attended school	18 (29.5)	60 (28.3)	Reference	0.96
Primary	40 (65.6)	139 (65.5)	0.95 (0.5, 1.8)	
Secondary	3 (4.9)	12 (5.7)	0.83 (0.2, 3.2)	
Vocational college/Tertiary	0 (0)	1 (0.5)	–	
Social economic status				
Lowest	15 (24.6)	43 (20.3)	Reference	0.03
Low	22 (36.0)	42 (19.8)	1.50 (0.6, 3.3)	
Middle	10 (16.4)	43 (20.3)	0.67 (0.3, 1.6)	
High	4 (6.6)	44 (20.7)	0.26 (0.1, 0.8)	
Highest	10 (16.4)	40 (18.9)	0.71 (0.3, 1.8)	
Alcohol drinking				
Never	31 (51.7)	77 (36.3)	Reference	0.06
Past	26 (43.3)	107 (50.5)	0.60 (0.3, 1.1)	
Current	3 (5.0)	28 (13.2)	0.27 (0.1, 0.9)	
Smoking status				
Never	45 (73.8)	152 (71.7)	Reference	0.92
Past	14 (22.9)	51 (24.1)	0.93 (0.5, 1.8)	
Current	2 (3.3)	9 (4.2)	0.75 (0.2, 3.6)	
Fruits and vegetables intake per day				
1–2 servings	26 (42.6)	55 (25.9)	Reference	0.04
3–4 servings	15 (24.6)	64 (30.2)	0.49 (0.2, 1.0)	
≥ 5 servings	20 (32.8)	93 (43.9)	0.45 (0.2, 0.9)	
Time spent in vigorous physical activity per week (minutes)				
Inadequate (<75 min)	13 (21.3)	51 (24.1)	Reference	0.66
Adequate (≥75 min)	48 (78.7)	161 (75.9)	1.17 (0.6, 2.3)	
Months on ART^c^	30 (4.5)	31 (5.2)	0.98 (09, 1.0)	0.57
Current ART regimen				
AZT + 3TC + EFV	9 (15.3)	32 (15.9)	Reference	0.92
AZT + 3TC + NVP	20 (33.9)	64 (31.8)	1.11 (0.5, 2.7)	
TDF + FTC + EFV	14 (23.7)	56 (27.9)	0.89 (0.3, 2.3)	
TDF + 3TC + EFV	16 (27.1)	49 (24.4)	1.16 (0.5, 2.9)	
TB treatment at baseline				
Did not receive TB treatment	52 (85.3)	158 (74.5)	Reference	0.08
Received TB treatment	9 (14.7)	54 (25.5)	0.50 (0.2, 1.1)	
TB treatment since baseline				
Did not receive TB treatment	50 (82.0)	146 (68.9)	Reference	0.048
Received TB treatment	11 (18.0)	66 (31.1)	0.48 (0.2, 0.9)	
Baseline CD4 count (cells/μL)	127 (104)	127 (98)	0.99 (0.9, 1.0)	0.99
Baseline CRP (Log -transformed)^d^	3.7 (1.7)	3.7 (1.8)	0.99 (0.8, 1.2)	0.91
Follow-up CRP (Log -transformed)	1.8 (1.6)	1.5 (1.4)	1.16 (0.9, 1.4)	0.14
NUSTART intervention assignment				
Control arm	30 (49.2)	98 (46.2)	Reference	0.68
Treatment arm	31 (50.8)	114 (53.8)	0.89 (0.5, 1.6)	
Baseline anthropometrics and body composition				
Waist circumference tertiles				
Lower	13 (21.3)	51 (24.1)	Reference	0.18
Middle	27 (44.3)	67 (31.6)	1.58 (0.7, 3.4)	
Upper	21 (34.4)	94 (44.3)	0.87 (0.4, 1.9)	
Hip circumference tertiles				
Lower	23 (37.7)	45 (21.2)	References	0.03
Middle	18 (29.5)	68 (32.1)	0.52 (0.3, 1.1)	
Upper	20 (32.8)	99 (46.7)	0.39 (0.2, 0.8)	
Body mass index tertiles				
Lower	16 (26.2)	50 (23.6)	Reference	0.78
Middle	21 (34.5)	68 (32.1)	0.96 (0.5, 2.0)	
Upper	24 (39.3)	94 (44.3)	0.79 (0.4, 1.6)	
Fat mass index tertiles^e^				
Lower	23 (41.8)	41 (21.2)	Reference	0.007
Middle	17 (30.9)	66 (34.2)	0.46 (0.2, 0.9)	
Upper	15 (27.3)	86 (44.6)	0.31 (0.1, 0.7)	
Fat-free mass index tertiles^e^				
Lower	18 (32.7)	64 (33.2)	Reference	0.67
Middle	17 (30.9)	70 (36.2)	0.86 (0.4,1.8)	
Upper	20 (36.4)	59 (30.6)	1.21 (0.6, 2.5)	
Follow-up anthropometrics and body composition				
Waist circumference tertiles				
Lower	32 (52.5)	61 (28.8)	Reference	0.0008
Middle	20 (32.8)	71 (33.5)	0.53 (0.3, 1.0)	
Upper	9 (14.7)	80 (37.7)	0.21 (0.1, 05)	
Hip circumference tertiles				
Lower	30 (49.2)	62 (29.3)	Reference	0.007
Middle	20 (32.8)	73 (34.4)	0.56 (0.3, 1.1)	
Upper	11 (18.0)	77 (36.3)	0.29 (0.1, 0.6)	
Body mass index tertiles				
Lower	30 (49.2)	61 (28.8)	Reference	0.009
Middle	18 (29.5)	73 (34.4)	0.50 (0.3, 0.9)	
Upper	13 (21.3)	78 (36.8)	0.34 (0.2, 0.7)	
Fat mass index tertiles^f^				
Lower	33 (55.0)	57 (27.3)	Reference	0.0003
Middle	17 (28.3)	73 (34.9)	0.40 (0.2, 0.8)	
Upper	10 (16.7)	79 (38.7)	0.21 (0.1, 0.5)	
Fat-free mass index tertiles^f^				
Lower	26 (43.3)	64 (30.6)	Reference	
Middle	13 (21.7)	77 (36.8)	0.41 (0.2, 0.9)	0.07
Upper	21 (35.0)	68 (32.6)	0.76 (0.4, 1.5)	

*ART* antiretroviral therapy, *AZT*, Zidovudine, *3TC* Lamivudine, *EFV* Efavirenz, *NVP*, Nevirapine, *TDF* Tenofovir, *FTC* Emtricitabine

^a^Patients with Pre-diabetes and diabetes

^b^Patients without pre-diabetes and diabetes

^c^PD/DM+ patients were 58 and PD/DM- patients were 194

^d^PD/DM+ patients were 53 and PD/DM- patients were 193

^e^PD/DM+ patients were 53 and PD/DM- patients were 193

^f^PD/DM+ patients were 60 and PD/DM- patients were 209

As seen in Table 4, in multiple regression models adjusted for age, sex, socioeconomic status, and baseline TB treatment, higher baseline hip circumference and fat mass index were associated with lower probability of having pre-diabetes and diabetes. At follow-up, the main predictor variables were adjusted for age, sex, socioeconomic status, history of TB treatment since baseline, alcohol drinking and vegetable and fruit intake. In these models, higher waist or hip circumference, BMI, fat mass index and fat-free mass index were associated with lower probabilities of having pre-diabetes and diabetes (*P* < 0.05 for all except hip circumference where *P* = 0.08). Baseline fat-free mass index was not associated with the study outcome, and baseline and follow-up CRP were not included in the multiple regressions because they were not predictors in univariable analysis. The results showed similar trends when body composition data were included as predictors in a continuous scale (Additional file 2: Table S2 and Additional file 3: Table S3) and additional analyses involving changes in anthropometric and body composition measurements as predictor variables ended with similar results as main analyses (Additional file 4: Table S4 and Additional file 5: Table S5).

**Table 4:** Multivariable analysis of anthropometric and body composition measurements as predictors for pre-diabetes and diabetes at 2 to 3 years post-ART

	Adjusted Odds Ratio (95% CI)	*P*-value
Baseline anthropometrics and body composition^a^		
Waist circumference tertiles		
Lower	Reference	0.19
Middle	1.49 (0.7, 3.3)	
Upper	0.79 (0.4, 1.8)	
Hip circumference tertiles		
Lower	Reference	0.05
Middle	0.57 (0.3, 1.2)	
Upper	0.41 (0.2, 0.8)	
Fat mass index tertiles		
Lower	Reference	0.002
Middle	0.30 (0.1, 0.7)	
Upper	0.20 (0.1, 0.5)	
Fat-free mass index tertiles		
Lower	Reference	0.73
Middle	0.79 (0.4,1.7)	
Upper	0.70 (0.3, 1.8)	
Follow-up anthropometrics and body composition^b^		
Waist circumference tertiles		
Lower	Reference	0.003
Middle	0.58 (0.3, 1.2)	
Upper	0.22 (0.1, 0.5)	
Hip circumference tertiles		
Lower	Reference	0.08
Middle	0.67 (0.3, 1.4)	
Upper	0.39 (0.2, 0.9)	
Body mass index tertiles		
Lower	Reference	0.01
Middle	0.46 (0.2, 1.00	
Upper	0.32 (0.1, 0.7)	
Fat mass index tertiles		
Lower	Reference	0.0008
Middle	0.39 (0.2, 0.8)	
Upper	0.19 (0.1, 0.5)	
Fat-free mass index tertiles		
Lower	Reference	0.05
Middle	0.36 (0.1, 0.8)	
Upper	0.62 (0.3, 1.3)	

^a^Analyses adjusted for age, sex, socio-economic status, and history of TB treatment at baseline

^b^Analyses adjusted for age, sex, socio-economic status, history of TB treatment since baseline, alcohol drinking, and vegetable and fruit intake

## Discussion

In this report we present results of follow-up evaluation of dysglycaemia among patients who were enrolled in the NUSTART trial 2–3 years earlier. Due to high mortality and loss to follow-up, we managed to assess 273 of 478 patients (57%) who were alive at trial conclusion (12 weeks post-ART initiation), which was 38% of 704 patients who were enrolled in the trial. In these patients, the prevalence of pre-diabetes and diabetes was 22%, and this was associated with lower fat mass at both ART referral and follow-up. Although frank diabetes was only about 1.5%, the prevalence of pre-diabetes comprising either impaired fasting glucose or impaired glucose tolerance was over 20%. Pre-diabetes is associated with an elevated risk of cardiovascular disease and of mortality [25–28], and in HIV-uninfected western populations 50% of patients with pre-diabetes develop diabetes in the span of 10 years or less [29]. Information as to whether this rapid progression from pre-diabetes to diabetes will be seen in resource-limited, high-HIV prevalence settings is lacking.

In this study, we hypothesized that excessive accumulation of adipose tissue occurring during ART on the background of high and persistent inflammation would be associated with pre-diabetes and diabetes. Although patients who were followed-up accumulated more adipose tissue relative to lean mass, it was patients who had lower baseline and follow-up fat mass rather than those with higher baseline and follow-up fat mass who were found to have pre-diabetes and diabetes. Unexpectedly, we did not see any effect of baseline or follow-up CRP on the study outcome in this followed-up cohort.

Several mechanisms may explain our unexpected findings. Since low fat mass or BMI is a marker of severe or prolonged HIV infection [30, 31], our results may suggest that pre-diabetes and diabetes occurred among malnourished patients who were severely immune-compromised or those with severe immune activation and that those who were less immune-compromised were spared. Patients with prolonged HIV immune activation, may have insulin resistance, and may subsequently develop pre-diabetes or diabetes mellitus [8]. However, we found no evidence that CRP (the marker of inflammation and immune activation used in this study) was associated with pre-diabetes and diabetes. Similarly, CD4 count (used to assess severity of HIV because HIV viral loads were not routinely measured in Tanzania) was not a predictor of pre-diabetes and diabetes. Another possibility is that we observed a variant of pre-diabetes and diabetes mellitus which is associated with nutritional deprivation rather than over nutrition. In Ethiopia, investigators found that compared to non-diabetes patients, diabetic patients receiving insulin were more likely to have low weight and height (if men) and low socioeconomic status and were more likely to have been undernourished during childhood than non-diabetics [32]. Similar findings have been reported in other settings [33, 34], and this variant of diabetes has been termed as malnutrition-related diabetes mellitus (MRDM). MRDM may occur in individuals with long standing malnutrition and is characterized by insulinopenia, insulin resistance and failure of beta-cell function resulting from irreversible pancreatic injury associated with pancreatic calculi formation [35]. Although there are little or no data on the effect of HIV-related prolonged undernutrition on diabetes risk, it is conceivable that severe nutritional depletion as occurred among patients who were eligible for the NUSTART trial could have led to pancreatic injury and consequently compromised beta-cell function leading to dysglycaemia presented in this study. Both Impaired Fasting Glucose (IFG) and Intolerance Glucose Tolerance (IGT) which are the basis for diagnosis of pre-diabetes and diabetes result from insulin resistance (hepatic and skeletal insulin resistance, respectively) and reduced insulin secretion [36–38]. Furthermore, since pre-diabetes and diabetes was associated with reduced rather than increased adipose tissue, it is likely that reduced insulin secretion due to MRDM rather than insulin resistance explains most the associations we present. However, detailed mechanistic studies are needed to explain well these observations.

The reported prevalence of pre-diabetes and diabetes in the current study is comparable with other reports of studies in Sub-Saharan Africa [39–41], but lower than what was recently reported in Mwanza by Maganga et al. [13]. In the latter study, investigators reported that the prevalence of pre-diabetes and diabetes among HIV-infected patients was 32.7% and that 18% of patients who had been on ART for at least 2 years had frank diabetes mellitus. We see at least one key methodological attribute which could explain the difference in these findings from the current study. The study population of Maganga et al. was drawn from the general HIV population with mean BMI at ART initiation of 22 kg/m^2^, whereas our study participants comprised individuals who started ART with BMI of <18.5 kg/m^2^, the level of BMI associated with higher mortality during ART [9]. Because the NUSTART participants who died before the current study had lower fat mass and BMI at ART initiation, and these are associated with increased risk of pre-diabetes and diabetes, it is possible that a higher proportion of deaths occurred among patients with pre-diabetes and diabetes compared to patients with normal glucose levels. This is plausible considering that high glucose level is also associated with elevated mortality [28, 42]. If this occurred, it would help explain a lower prevalence of pre-diabetes and diabetes among patients we followed-up in comparison to the recent report [13]. Irrespective of reasons explaining differences in these results, it is clear that the burden of pre-diabetes and diabetes among HIV-infected patients in Tanzania is very high and the Tanzanian Ministry of Health should develop strategies to reduce diabetes risk among patients on ART and provide appropriate care to those developing frank diabetes to reduce the risk of mortality and the complications of diabetes. Other countries in the region may be similarly affected, and should consider prevention, screening, and treatment programs as well.

The prevalences of most traditional diabetes risk factors were low in our study population. The prevalence of overweight and obesity was only 4.4%, and that of current smoking was 4%. In addition, more than three quarters of patients spent more than 75 min in vigorous physical activity per week, which is an adequate level of physical activity according to WHO [43]; all patients had more than the WHO recommended level of moderate activity, so this was not included as a risk factor in analyses. However, we found that only 41% of study participants had adequate intake of fruit and vegetables. Although intake of fruits and vegetables was not a predictor of pre-diabetes and diabetes in multivariable analysis (data not shown), promotion of fruit and vegetable intake among HIV-infected patients should be included in chronic disease reduction programs as it might help to reduce the risk of other non-communicable diseases related to diabetes in these patients [44, 45]. CD4 count at referral to ART and use of particular ART regimens were not associated with pre-diabetes and diabetes.

Regarding socio-economic status, we found that lower socioeconomic class seemed to be significantly associated with higher risk of pre-diabetes and diabetes in unadjusted analysis.

This is similar to findings in other African settings. In Ethiopia, investigators found that diabetes patients were more likely to have lower socioeconomic status than non-diabetics, and in poor urban Kenyan communities the prevalence of diabetes was 5% [46], the level comparable with prevalence in the general population in SSA [1]. The popular belief in some SSA settings is that diabetes is the disease of people from high social classes because they are prone to diabetes risk factors like obesity and physical inactivity unlike those in low social economic class. By contrast, findings from this study and previous reports suggest that the prevalence of non-communicable diseases in poor people including those with HIV in SSA is likely to be higher, and efforts to control these diseases should not neglect these populations.

The main limitation of the study was that it was conducted among patients who had BMI less than 18.5 (kg/m^2^) at ART initiation, so it may not be possible to extrapolate our results to those not malnourished at the start of ART, but this does not reduce the external validity of our findings since a sizeable proportion of patients starting ART in Tanzania are undernourished [9]. In the present study we were not able to follow-up 58.1% of 704 patients enrolled in NUSTART trial. Of those not seen 66% (286) had died before follow-up and 34% (145) were not traceable, had declined to participate or had travelled. Those not traceable, declined or travelled were similar in baseline body mass index and fat mass index as those followed-up and therefore are likely to have the same prevalence of pre-diabetes or diabetes as those followed-up. However, those died had lower body mass index and fat mass index. As explained above, since low body mass index and fat mass index are predictors of of pre-diabetes and diabetes in this cohort it is possible that those died had higher prevalence of glucose dysglycemia than the cohort followed-up. Therefore, the prevalence of pre-diabetes and diabetes we report may be an underestimate of the true burden of dysglycemia among undernourished HIV patients at ART initiation in this setting.

This was a cross-sectional study, so it is difficult to conclude the directionality of predictors and study outcome, although it is unlikely that pre-diabetes should lead to low fat reserve. Moreover, we did not have an HIV-negative control group or an HIV-infected but ART naive group which would have helped to disentangle the role of HIV and ART in the occurrence of pre-diabetes and diabetes in this population.

Regarding predictors, we found CRP and CD4 count were not predictors of pre-diabetes and diabetes. However, CRP is non-specific marker of inflammation and CD4 counts may not be a good marker HIV progression in malnourished patients [47]. Future studies should explore the association of inflammation with pre-diabetes and diabetes using more specific inflammatory markers like Interleukin 6 (IL-6) and use viral load as marker of severity of HIV infection in assessing predictors of dysglycemia among HIV patients.

## Conclusions

In this study we found that in HIV-infected adults on ART who were malnourished at ART initiation 2–3 years earlier, low adiposity at both baseline and follow-up were associated with increased likelihood of having pre-diabetes and diabetes. Surprisingly, CRP was not a predictor. Further studies in general HIV-infected populations are needed to confirm these findings and to further investigate the role of body composition, HIV, ART, and inflammation in the pathogenesis of diabetes among HIV-infected populations in order to improve the evidence base for developing interventions to reduce diabetes risk in this population.

## Additional files

Additional file 1: Table S1. Comparison of body composition and clinical presentation of patients with and without pre-diabetes and diabetes at 2-3 years post-ART initiation^1^. (DOC 36 kb)
Additional file 2: Table S2. Univariate analysis of anthropometric and body composition measurements as predictors for pre-diabetes and diabetes at 2 to 3 years post-ART. (DOC 36 kb)
Additional file 3: Table S3. Multivariable analysis of anthropometric and body composition measurements as predictors for pre-diabetes and diabetes at 2 to 3 years post-ART. (DOC 34 kb)
Additional file 4: Table S4. Univariate analysis of changes in anthropometric and body composition measurements as predictors for pre-diabetes and diabetes at 2 to 3 years post-ART. (DOC 32 kb)
Additional file 5: Table S5. Multivariable analysis of changes in anthropometric and body composition measurements as predictors for pre-diabetes and diabetes at 2 to 3 years post-ART. (DOC 30 kb)

## Abbreviations

ART: Antiretroviral therapy
BMI: Body mass index
CRP: C-Reactive protein
HIV: Human immune deficiency virus
IFG: Impaired fasting glucose
IGT: impaired glucose tolerance
IL-6: Interleukin-6
NIMR: National Institute for Medical Research
NUSTART: Nutritional support for africans starting antiretroviral therapy
OGTT: Oral glucose tolerance test
SSA: Sub-Saharan Africa
TB: Tuberculosis
TNF: Tumour necrosis factor
WHO: World Health Organization
