# Supplementary material for: Dysglycemia associations with adipose tissue among HIV-infected patients after 2 years of antiretroviral therapy in Mwanza: a follow-up cross-sectional study
Source: BMC Infect Dis. 2017 Jan 30;17:103. doi: 10.1186/s12879-017-2209-z (PMC5282875; doi:10.1186/s12879-017-2209-z)
Supplement: Additional file 1: Table S1. — Comparison of body composition and clinical presentation of patients with and without pre-diabetes and diabetes at 2-3 years post-ART initiation1. (DOC 36 kb) [file 12879_2017_2209_MOESM1_ESM.doc]

| Additional file 1: Table S1 Comparison of body composition and clinical presentation of patients with and without pre-diabetes and diabetes at 2-3 years post-ART initiation^1^ | | | | |
| --- | --- | --- | --- | --- |
|  | Patients with pre-diabetes and diabetes (n=61) | Patients with no pre-diabetes and diabetes (n=212) | Mean difference (95% CI) | *P* value^2^ |
|  | Mean (sd) or n (%) | Mean (sd) or n (%) |  |  |
| Waist circumference (cm) | 73.0 (5.1) | 76.9 (7.5) | 3.9 (1.9-5.9) | 0.0002 |
| Hip circumference (cm) | 85.2 (5.7) | 89.5 (7.0) | 4.2 (2.3, 6.2) | <0.0001 |
| Weight (kg) | 49.8 (7.0) | 53.7 (8.6) | 3.9 (1.5,6.3) | 0.002 |
| Body mass index(kg/m^2^) | 19.9 (2.1) | 20.2 (2.9) | 1.2 (0.4,2.0) | 0.002 |
| Fat mass index (kg/m^2^)^3^ | 3.2 (1.7) | 4.5 (2.5) | 1.3 (0.6, 1.9) | 0.0002 |
| Fat-free mass index (kg/m^2^)^3^ | 15.6 (1.6) | 15.5 (1.5) | -0.06 (-0.5, 0.4) | 0.79 |
| Excessive urination | 16 (26.2) | 62 (29.3) |  | 0.65 |
| Excessive water drinking | 17 (27.9) | 59 (27.8) |  | 0.99 |
| Excessive hunger | 16 (26.2) | 69 (32.6) |  | 0.35 |
| Loss of weight | 27 (44.3) | 80 (37.7) |  | 0.36 |
| Tiredness | 22 (36.1) | 68 (32.1) |  | 0.56 |
| Numbness of limbs | 17 (27.9) | 78 (36.8) |  | 0.20 |
| Poor vision | 20 (32.8) | 64 (30.2) |  | 0.70 |
| Frequent ulcers | 7 (11.5) | 25 (11.8) |  | 0.95 |
| Frequent infections | 11 (18.0) | 33 (15.6) |  | 0.64 |
| ^1^Data are mean (sd) or n (%) ^2^comparison done by chi-squared test or ttest ^3^60 patients in pre-diabetes and diabetes group and 209 patients with no pre-diabetes or diabetes included in analysis | | | | |
