# Supplementary material for: Dysglycemia associations with adipose tissue among HIV-infected patients after 2 years of antiretroviral therapy in Mwanza: a follow-up cross-sectional study
Source: BMC Infect Dis. 2017 Jan 30;17:103. doi: 10.1186/s12879-017-2209-z (PMC5282875; doi:10.1186/s12879-017-2209-z)
Supplement: Additional file 2: Table S2. — Univariate analysis of anthropometric and body composition measurements as predictors for pre-diabetes and diabetes at 2 to 3 years post-ART. (DOC 36 kb) [file 12879_2017_2209_MOESM2_ESM.doc]

| Additional file 2: Table S2 Univariate analysis of anthropometric and body composition measurements as predictors for pre-diabetes and diabetes at 2 to 3 years post-ART | | | | |
| --- | --- | --- | --- | --- |
|  | PD/DM+^1^  (n=61) | PD/DM-^2^  (n=212) | Odds Ratio  (95% CI) | *P*-*value* |
|  | mean (sd) | mean (sd) |  |  |
| Baseline anthropometrics and body composition |  |  |  |  |
| Waist circumference (cm) | 66.9 (4.2) | 67.8 (4.7) | 0.95 (0.9, 1.0) | 0.17 |
| Hip circumference (cm) | 79.0 (4.3) | 80.7 (4.1) | 0.90 (0.8, 0.9) | 0.005 |
| Body mass index (kg/m^2^) | 16.6 (1.2) | 16.8 (1.2) | 0.91 (0.7, 1.1) | 0.45 |
| fat mass index (kg/m^2^)*^3^* | 2.6 (0.7) | 2.9 (0.8) | 0.62 (0.4, 0.9) | 0.02 |
| Fat-free mass index (kg/m^2^)*^3^* | 14.1 (1.0) | 14.0 (0.8) | 1.19 (0.8, 1.7) | 0.32 |
| Follow-up anthropometrics and body composition |  |  |  |  |
| Waist circumference (cm) | 73.0 (5.1) | 76.9 (7.4) | 0.90 (0.8, 0.9) | <0.0001 |
| Hip circumference (cm) | 85.2 (5.7) | 89.5 (7.0) | 0.89 (0.8, 0.9) | <0.0001 |
| Body mass index (kg/m^2^) | 19.0 (2.1) | 20.2 (2.9) | 0.80 (0.7, 0.9) | 0.002 |
| Fat mass index (kg/m^2^)^4^ | 4.2 (1.3) | 5.1 (1.8) | 0.65 (0.5, 0.8) | <0.0001 |
| Fat-free mass index(kg/m^2^)*^4^* | 14.8 (1.1) | 15.2 (1.2) | 0.77 (0.6, 1.0) | 0.06 |
| ^1^Patients with Pre-diabetes and diabetes; ^2^Patients without pre-diabetes and diabetes; ^3^PD/DM+ patients were 53 and PD/DM- patients were 193; ^4^PD/DM+ patients were 60 and PD/DM- patients were 209 | | | | |
