# Supplementary material for: Dysglycemia associations with adipose tissue among HIV-infected patients after 2 years of antiretroviral therapy in Mwanza: a follow-up cross-sectional study
Source: BMC Infect Dis. 2017 Jan 30;17:103. doi: 10.1186/s12879-017-2209-z (PMC5282875; doi:10.1186/s12879-017-2209-z)
Supplement: Additional file 4: Table S4. — Univariate analysis of changes in anthropometric and body composition measurements as predictors for pre-diabetes and diabetes at 2 to 3 years post-ART. (DOC 32 kb) [file 12879_2017_2209_MOESM4_ESM.doc]

| Additional file 4: Table S4 Univariate analysis of changes in anthropometric and body composition measurements as predictors for pre-diabetes and diabetes at 2 to 3 years post-ART | | | | |
| --- | --- | --- | --- | --- |
|  | PD/DM+^1^  (n=61) | PD/DM-^2^  (n=212) | Odds Ratio  (95% CI) | *P*-*value* |
|  | n or mean (sd) | n or mean (sd) |  |  |
| Anthropometric and body composition changes from baseline to 2-3 years post-ART |  |  |  |  |
| Waist circumference (cm) | 6.1 (5.6) | 9.0 (7.9) | 0.93 (0.8, 0.9) | 0.007 |
| Hip circumference (cm) | 6.2 (5.8) | 8.8 (7.0) | 0.90 (0.8, 0.9) | 0.01 |
| Body mass index (kg/m^2^) | 2.3 (2.0) | 3.4 (2.9) | 0.82 (0.7, 0.9) | 0.005 |
| Fat mass index (kg/m^2^)^3^ | 1.5 (1.2) | 2.2 (1.8) | 0.74 (0.6, 0.9) | 0.02 |
| Fat-free mass index (kg/m^2^)*^3^* | 0.7 (0.9) | 1.1 (1.1) | 0.70 (0.5, 0.9) | 0.02 |
| ^1^Patients with Pre-diabetes and diabetes ^2^Patients without pre-diabetes and diabetes ^3^PD/DM+ patients were 55 and PD/DM- patients were 193 | | | | |
