# Supplementary material for: Dysglycemia associations with adipose tissue among HIV-infected patients after 2 years of antiretroviral therapy in Mwanza: a follow-up cross-sectional study
Source: BMC Infect Dis. 2017 Jan 30;17:103. doi: 10.1186/s12879-017-2209-z (PMC5282875; doi:10.1186/s12879-017-2209-z)
Supplement: Additional file 5: Table S5. — Multivariable analysis of changes in anthropometric and body composition measurements as predictors for pre-diabetes and diabetes at 2 to 3 years post-ART. (DOC 30 kb) [file 12879_2017_2209_MOESM5_ESM.doc]

| Additional file 5: Table S5 Multivariable analysis of changes in anthropometric and body composition measurements as predictors for pre-diabetes and diabetes at 2 to 3 years post-ART | | |
| --- | --- | --- |
|  | Odds Ratio (95% CI) | *P*-*value* |
| Anthropometric and body composition changes from baseline to 2-3 years post-ART^1^ |  |  |
| Waist circumference (cm) | 0.93 (0.8, 0.9) | 0.02 |
| Hip circumference (cm) | 0.95 (0.9 1.0) | 0.11 |
| Body mass index (kg/m^2^) | 0.82 (0.7, 0.9) | 0.02 |
| Fat mass index (kg/m^2^) | 0.78 (0.6, 1.0) | 0.08 |
| Fat-free mass index (kg/m^2^) | 0.71 (0.5, 1.0) | 0.06 |
| ^1^Analyses adjusted for age, sex, socio-economic status, history of TB treatment since baseline, alcohol drinking, and vegetable and fruit intake | | |
